# ^18^F-FDG PET/CT in the diagnosis and treatment of atypical extensive skin lesions in extranodal natural killer/T-cell lymphoma, nasal type: a case report

**DOI:** 10.3389/fonc.2025.1480661

**Published:** 2025-04-01

**Authors:** Xu-Sheng Liu, Zhi-Jun Pei

**Affiliations:** Department of Nuclear Medicine, Taihe Hospital, Hubei University of Medicine, Shiyan, Hubei, China

**Keywords:** lymphoma, nasal type, ^18^F-FDG, PET/CT, therapy

## Abstract

We report a case of a 60-year-old man who developed scattered erythema and papules on his left upper limb without any apparent cause 6 months ago. Initially, the patient underwent evaluations for various dermatological conditions, including eczema and psoriasis, while also being assessed for potential malignancies such as cutaneous lymphoma or sarcoidosis, but no definitive diagnosis was made. Over time, the patient’s symptoms progressed, presenting as generalized erythema, papules, localized itching, and pain. A histopathological examination of the lesions diagnosed it as extranodal NK/T-cell lymphoma, nasal type (ENKTCL-NT). Subsequently, the patient was referred for staging via 18F-fluorodeoxyglucose positron emission tomography/computed tomography (^18^F-FDG PET/CT), which revealed a widespread pattern of metabolically active lesions primarily affecting the skin and subcutaneous tissue of the limbs and buttocks. The patient received sequential treatment with P-GEMOX (gemcitabine, oxaliplatin, and pegaspargase) and underwent clinical examination and follow-up whole-body ^18^F-FDG PET/CT after six treatment cycles. The post-treatment PET/CT showed no abnormal ^18^F-FDG uptake in the nasal cavity or skin, confirming clinical complete remission. Our case highlights the significant role of ^18^F-FDG PET/CT in clinical practice for initial staging and treatment response assessment of ENKTCL-NT.

## 1 Introduction

Natural killer/T-cell lymphoma (NKTL) is a subtype of Epstein-Barr virus (EBV)-related non-Hodgkin lymphoma. More than two-thirds of NKTL lymphomas are localized at diagnosis and often receive radiation therapy alone, with a 5-year survival rate of approximately 70% (1–3). However, a subset of NKTL, known as extranodal NK/T-cell lymphoma, nasal type (ENKTCL-NT), stands out for its rapid progression, invasive growth patterns, and severe symptomatology, which contribute to a particularly poor prognosis. ENKTCL-NT is rare but poses significant therapeutic challenges due to its aggressive nature (4–6). The typical clinical manifestations of ENKTCL-NT include necrosis affecting the nasal cavity or upper respiratory and digestive structures. Extranodal lesions, such as those on the skin and intestinal mucosa, are less common (2, 5). Due to its atypical presentation and limited existing research, this condition poses unique challenges for diagnosis and treatment. The role of 18F-fluorodeoxyglucose positron emission tomography/computed tomography (^18^F-FDG PET/CT) imaging in the diagnostic, staging, and prognostic assessment of ENKTCL-NT has been well-documented in the literature. This imaging modality provides critical insights into the metabolic activity of tumors, which can be pivotal in guiding clinical management (7–9). This case report highlights the effectiveness of ^18^F-FDG PET/CT in thoroughly evaluating and assessing the response of ENKTCL-NT.

## 2 Case presentation

**Figure 1:**
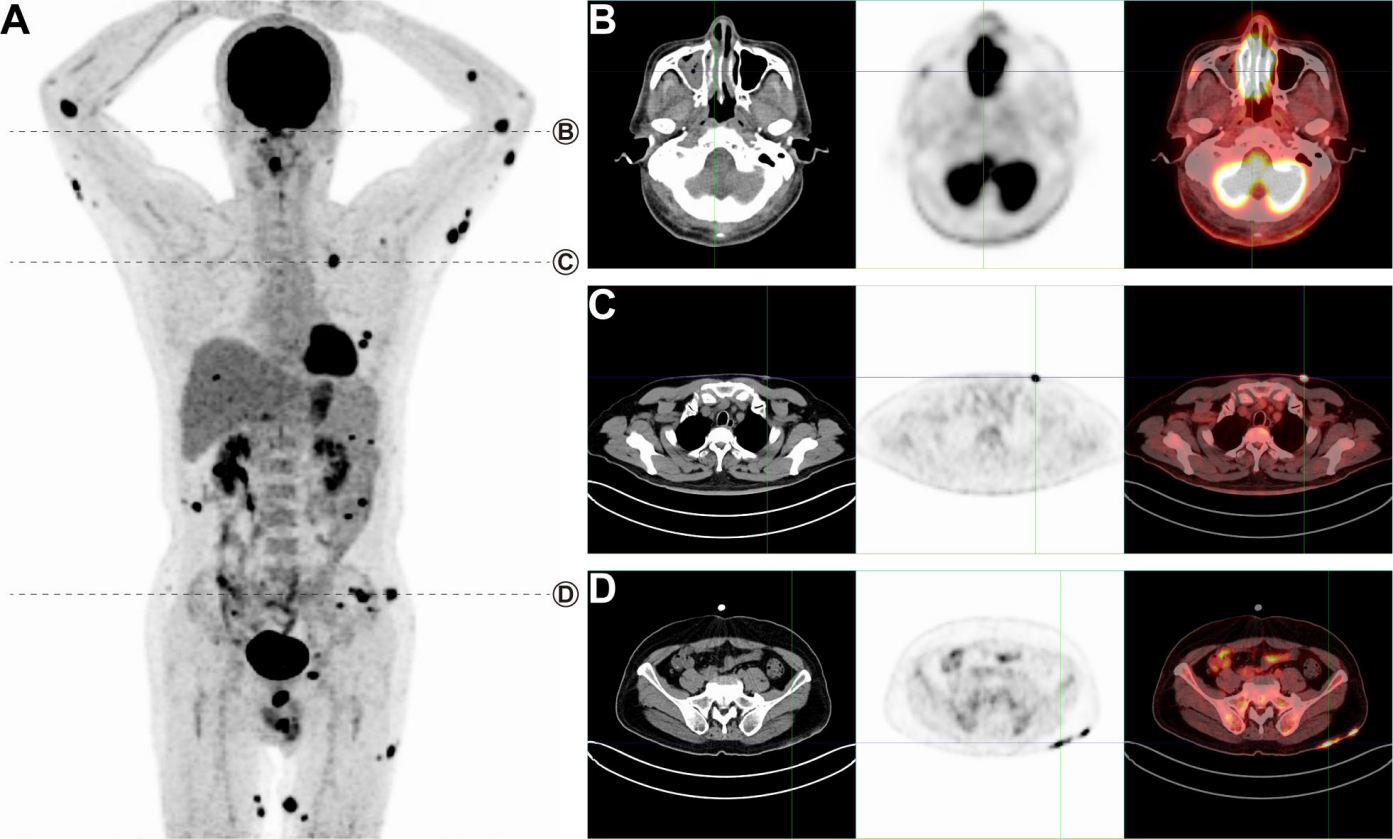
PET/CT imaging before treatment demonstrating extensive FDG uptake in multiple regions, indicating active disease involvement. **(A)** A maximum intensity projection (MIP) whole-body image shows widespread hypermetabolic lesions. **(B)** Significant ^18^F-FDG uptake in the nasal cavity (SUVmax 27.6) suggests aggressive disease in the nasal region. **(C)** Lesions on the left chest wall (SUVmax 24.1) and **(D)** left buttock (SUVmax 15.7) also demonstrate intense FDG uptake, indicative of extranasal disease spread.

**Figure 2:**
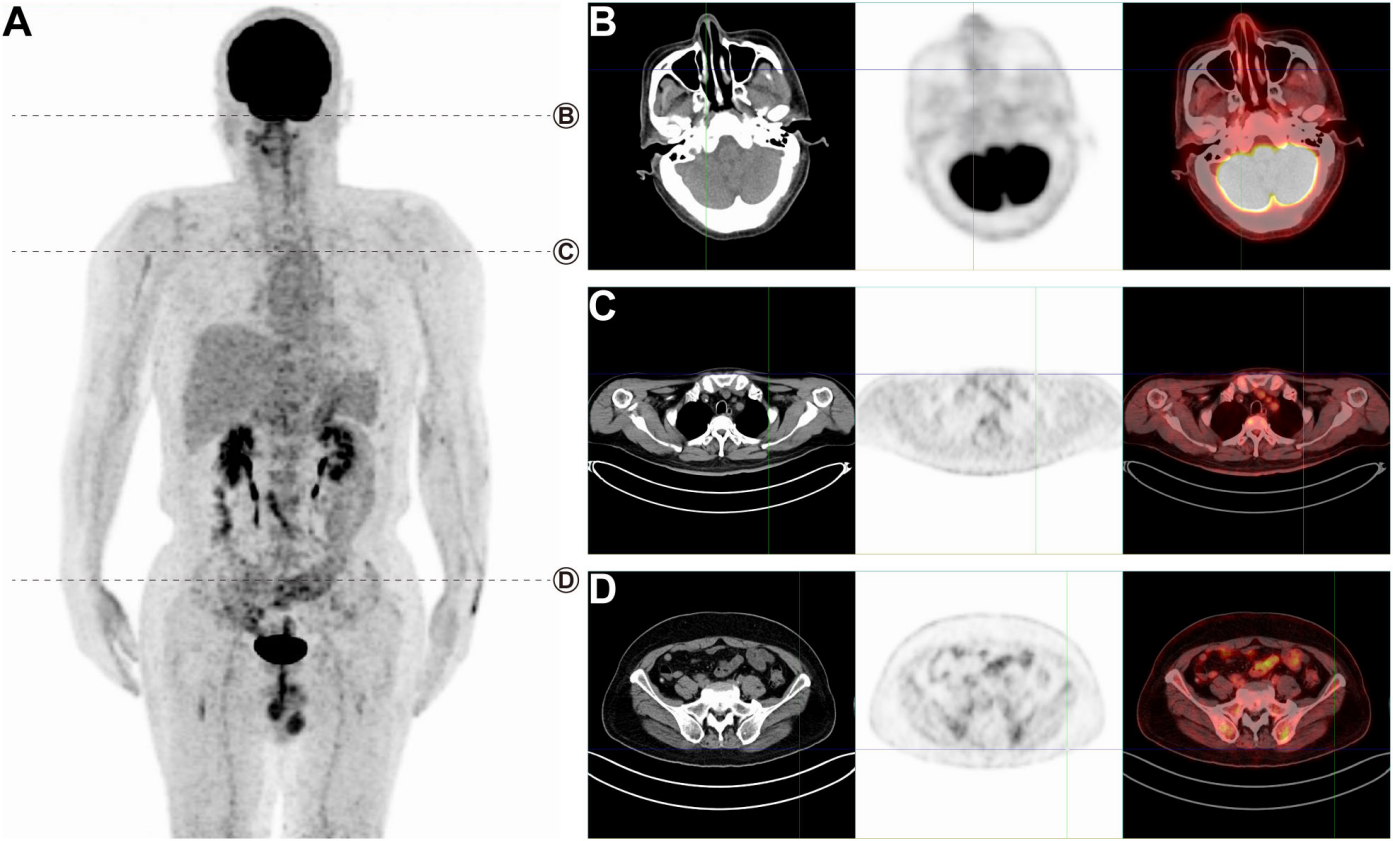
PET/CT imaging after treatment. **(A)** A maximum intensity projection (MIP) whole-body image. **(B–D)** No abnormal uptake from the nasal cavity or skin.

We present the case of a 60-year-old man who initially complained of scattered erythema and papules on his left upper limb, which had appeared without any identifiable cause 6 months prior to presentation. The patient was first evaluated for dermatological conditions, including eczema and psoriasis, and other potential malignancies, such as cutaneous lymphoma or sarcoidosis. Over time, the patient experienced a progression of symptoms, including generalized erythema, papules, localized pruritus, and pain. Concurrently, he reported nasal congestion, episodes of dizziness, and significant weight loss. Upon histopathological examination, the patient’s lesions were found to exhibit lymphocyte hyperplasia with infiltration into the subcutaneous fat lobules. Immunohistochemical analysis revealed a positive profile for CD3, CD4, CD8, CD56, perforin, granzyme B, TIA-1, EBER, and a high Ki-67 index (80%). Based on the morphological findings and immunohistochemical staining, the diagnosis of ENKTCL-NT was established. The patient was subsequently referred for staging with 18F-FDG PET/CT, which revealed a widespread pattern of metabolically active lesions, particularly affecting the skin and subcutaneous tissues of the limbs and buttocks (**Figure 1A**, SUVmax 15.7 to 27.6). In contrast, ENKTCL-NT cases involving only the nasal cavity and no distant metastases tend to have typical SUVmax values between 3 and 5 (10), indicating that metabolic activity was more aggressive in the context of extensive skin involvement. Notably, there was also increased 18F-FDG uptake in the nasal cavity (**Figure 1B**, SUVmax 27.6), indicative of disease involvement at this site. Furthermore, the PET/CT scan showed significant ^18^F-FDG uptake in lesions in the left chest wall (**Figure 1C**, SUVmax 24.1) and left buttock (**Figure 1D**, SUVmax 15.7). Typical lesions of ENKTCL-NT mainly affect the nose, upper respiratory tract, and digestive structures and rarely involve extranasal sites. However, this patient presented with extensive skin lesions. Based on clinical data and PET/CT scan results, the clinical diagnosis of NKTL stage IVB was made. The patient received sequential therapy with P-GEMOX (6) for six cycles, specifically gemcitabine (1,000 mg/m², intravenous infusion on days 1 and 8), oxaliplatin (100 mg/m², intravenous infusion on day 1), and pegaspargase (2,000 U/m², intramuscular injection on day 1), with each cycle lasting 3 weeks. A clinical examination and follow-up whole-body ^18^F-FDG PET/CT were performed after completing the six treatment cycles (**Figure 2A**). The post-treatment PET/CT demonstrated no abnormal ^18^F-FDG uptake in the nasal cavity or skin, confirming a complete clinical remission (**Figures 2B–D**).

## 3 Discussion

ENKTCL-NT is a rare and aggressive subtype of NHL, predominantly affecting the nasal cavity and upper respiratory tract structures. Despite advancements in treatment strategies, ENKTCL-NT remains a therapeutic challenge, particularly in cases of advanced or relapsed disease (4, 5). The prognosis for these patients is often poor, highlighting the need for effective diagnostic and prognostic tools.

^18^F-FDG PET/CT is a powerful diagnostic tool that combines the functional imaging capabilities of PET with the anatomical detail provided by CT. This technique relies on the uptake of ^18^F-FDG, a glucose analog, by tumor cells, allowing for the assessment of glucose metabolism and, by extension, tumor activity. In the context of ENKTCL-NT, ^18^F-FDG PET/CT has been shown to be particularly useful in determining tumor location, extent, and distribution, thereby complementing other imaging modalities and enhancing diagnostic accuracy (7, 9). A prospective study by Xu et al. has demonstrated the prognostic value of 18F-FDG PET/CT in ENKTCL-NT, suggesting that this imaging technique can provide valuable insights into patient outcomes (8).

Typical manifestations of ENKTCL-NT include necrotic processes affecting the nose or upper respiratory tract and digestive structures and rarely involve extranasal sites (5). However, cases with esophageal involvement (11) and primary muscle involvement (12) have been reported, indicating the need for a high index of suspicion for atypical presentations. In the case described by Zhang (11), esophageal lymphoma presented with segmentally increased FDG uptake, mimicking esophagitis, which underscores the importance of considering lymphoma in the differential diagnosis of hypermetabolic esophageal diseases.

Recurrence of ENKTCL-NT can also occur in unusual locations, as seen in the case reported by Yue Zhang (13), where the disease recurred at the penile glans, initially suspected to be urinary contamination on 18F-FDG PET/CT. This case highlights the diagnostic challenges in identifying disease recurrence, especially in atypical locations, and the importance of a thorough clinical evaluation.

In the treatment of ENKTCL-NT, chemotherapy is the dominant treatment (6), particularly for advanced-stage or extranasal disease. The P-GEMOX regimen (gemcitabine, oxaliplatin, and pegaspargase) was chosen for our patient due to its efficacy in treating ENKTCL-NT with extensive disease (14). While radiation is often used for localized disease confined to the nasal region, chemotherapy becomes the treatment of choice for more advanced stages (IVB), especially when skin involvement is present. Other treatment regimens, such as SMILE (steroids, methotrexate, ifosfamide, L-asparaginase, and etoposide), could also be considered, but they often come with higher toxicity profiles. We did not observe significant adverse effects during the course of the P-GEMOX regimen in this patient, and a follow-up PET/CT scan after six treatment cycles confirmed complete remission. The lack of abnormal FDG uptake in the post-treatment scan (**Figure 2**) further underscores the efficacy of this chemotherapy regimen in achieving metabolic remission.

The prognosis for patients with ENKTCL-NT, especially those with atypical extranasal sites, such as the skin, remains poor. However, as illustrated in this case, early and aggressive treatment can yield favorable outcomes. Luo et al. (15) investigated the prognostic utility of radiomics features in 18F-FDG PET/CT in predicting progression-free survival (PFS) and overall survival (OS) in ENKTCL patients. They found that a clinical, metabolic, and radiomics model had the greatest discriminative ability for both PFS and OS, suggesting that PET/CT-based radiomics could be a valuable tool for risk stratification and prognostication in clinical practice.

The current guidelines emphasize the importance of close follow-up through PET/CT imaging every 3 to 6 months for the first 2 years post-treatment due to the significant risk of relapse. In this instance, we plan to conduct serial PET/CT scans at regular intervals to monitor for potential disease recurrence. The presence of extensive skin lesions in ENKTCL-NT is rare and has been the subject of limited clinical research. Our case highlights the importance of ^18^F-FDG PET/CT in providing quantitative measurements of ^18^F-FDG uptake and metabolic rate, which can serve as objective metrics for assessing disease extent and treatment response. These metrics are crucial for the selection of appropriate treatment strategies, adjustment of treatment regimens, and monitoring of therapeutic efficacy.

## 4 Conclusion

In summary, this case report highlights the pivotal role of 18F-FDG PET/CT in evaluating and managing ENKTCL-NT, particularly in rare cases with extensive skin lesions. PET/CT proved essential in detecting widespread disease, monitoring treatment response, and achieving complete remission in this patient. Given the rarity and aggressiveness of skin-involved ENKTCL-NT, the case underscores the value of individualized, PET/CT-guided treatment approaches. This imaging technique enhances diagnostic precision and optimizes outcomes, as demonstrated by the patient’s successful remission. As research advances, 18F-FDG PET/CT is poised to play an increasingly vital role in managing ENKTCL-NT and other aggressive lymphomas.

## Data Availability

The original contributions presented in the study are included in the article/supplementary material. Further inquiries can be directed to the corresponding author.
